# Diarrhoeagenic *Escherichia coli* isolated from children with acute diarrhoea at Rakai hospital, Southern Uganda

**DOI:** 10.4314/ahs.v22i1.67

**Published:** 2022-03

**Authors:** Fredrick Masiga, Edgar Kigozi, Christine Florence Najjuka, Henry Kajumbula, David Patrick Kateete

**Affiliations:** 1 Makerere University Hospital, Kampala, Uganda; 2 Department of Medical Microbiology, School of Biomedical Sciences, Makerere. University College of Health Sciences, Kampala, Uganda; 3 Department of Immunology and Molecular Biology, School of Biomedical Sciences, Makerere University College of Health Sciences, Kampala, Uganda

**Keywords:** Childhood diarrhea, *Escherichia coli*, Diarrhoeagenic *Escherichia coli* (DEC), Enteroaggregative *Escherichia coli* (EAEC), Enteropathogenic *Escherichia coli* (EPEC), Enterotoxigenic *Escherichia coli* (ETEC), Enteroinvasive *Escherichia coli* (EIEC), Enterohemorrhagic *Escherichia coli* (EHEC), Rakai General Hospital, Uganda

## Abstract

**Background:**

Diarrhoeagenic *Escherichia coli* (DEC) is a leading cause of childhood diarrhoea. This study estimated the prevalence of DEC and DEC pathotypes among children with acute diarrhoea in Southern Uganda.

**Methods:**

A cross-sectional study was conducted on 267 children less than 5 years with acute diarrhoea, admitted to Rakai General Hospital in Southern Uganda. Faecal samples were collected from the children and processed for isolation of *E. coli*. The presence of DEC and the distribution of DEC pathotypes were determined by polymerase chain reaction.

**Results:**

A total of 102 (38.2%, 102/267) children had DEC of various pathotypes – enteroaggregative *E. coli* (EAEC) (14.2%); enteropathogenic E. coli (EPEC) (6.7%); enterotoxigenic *E. coli* (ETEC) (6%); enteroinvasive *E. coli* (EIEC) (7.5%); enterohemorrhagic *E. coli* (EHEC) (3%); and cell-detaching E. coli (CDEC) (0.75%). The difference in the overall prevalence of DEC was not significant regarding HIV but individually, EAEC and CDEC were associated with HIV-positive status while ETEC was associated with HIV-negative status.

**Conclusions:**

DEC is prevalent in children with acute diarrhoea in Southern Uganda and its identification in children should be considered among strategies for combatting childhood diarrhoea in Africa.

## Background

Diarrhoea is a leading cause of morbidity and mortality in children less than 5 years and it is a common problem in HIV-infected individuals worldwide 1, 2. While tried-andtested interventions have shown that diarrheal deaths are preventable 3, diarrhoea remains a leading cause of death in Africa, and in 2015, it was responsible for ∼330,000 deaths in children less than 5 years 4.

Diarrhoeagenic *Escherichia coli* (DEC) is one of the major causes of gastrointestinal disorders worldwide 5–8. DEC is classified into six major pathotypes (pathogenic variants) depicting the disorders associated with each pathotype i.e. enteroaggregative *E. coli* (EAEC), enteropathogenic *E. coli* (EPEC), enterotoxigenic E. coli (ETEC), enteroinvasive *E. coli* (EIEC), enterohaemorrhagic *E. coli* (EHEC also known as Shiga-toxin producing *E. coli*), and diffusely adherent *E. coli* (DAEC) 6, 9–11. Although E. coli is commonly associated with acute diarrhoea in children in Uganda 12, the prevalence of DEC and its pathotypes has not been documented. The aim of this study was to determine the frequency of DEC and the distribution of DEC pathotypes among children presenting with acute diarrhoea admitted to Rakai General Hospital in Southern Uganda. We also aimed to compare the prevalence of DEC and the distribution of DEC pathotypes among HIV-positive vs. HIV-negative children with acute diarrhoea.

## Methods

This was a cross-sectional study of children aged 6 months to 5 years admitted to Rakai General Hospital in Southern Uganda. Rakai Hospital is a 100-bed capacity hospital that admits approx. 200 sick children every week. The study recruited children with acute diarrhoea (passage of 3 or more loose stool over a 24-hour period lasting less than 2 weeks). HIV-serostatus was performed according to the algorithm of Uganda's Ministry of Health. Stool was obtained from each child by using a sterile plastic container labelled with a unique study number. Specimen containers and instructions pertaining specimen collection were given to caretakers of the recruited cildren. For younger children, mothers collected stool from diapers as soon as the children passed the stool while for older children, stool was collected into a disposable plate and immediately transferred into a sterile container. As soon as specimens were obtained, the caretakers were instructed to deliver them to Kakuuto Health Centre IV Microbiology Laboratory, Rakai district where they were processed for growth and identification of E. coli. In the laboratory, each sample was inoculated onto a MacConkey agar plate within an hour of reception and incubated at 37°C for 14 hours. Lactose fermenting colonies suggestive of *E. coli* were inoculated onto IMViCU (Indole, Methyl red, Voges Proskaeur, Citrate and Urease) medium for confirmation as *E. coli*. Isolates that were positive with indole and methyl red tests but negative with Voges-Proskaeur, citrate and urea were classified as *E. coli*. To increase chances of detecting DEC, five *E. coli* colonies were screened from each plate. Isolates were separately preserved in tryptone soy broth and transported to Makerere University for molecular analysis.

Crude and/or pure chromosomal DNA used in the polymerase chain reaction (PCR) was extracted as described previously 13. Briefly, each sample was inoculated onto nutrient agar and incubated at 37°C for 24 hours. To extract the DNA, a swipe with colonies was suspended into 300 µl of sterile 0.25X TE (Tris-EDTA) buffer in 1.5 ml microcentrifuge tubes, vortexed for 10 seconds and centrifuged at 13,000 g for 2 minutes. Then, the supernatant was discarded to retain the pellet of clean bacteria, to which we added 100 µl of 0.25X TE buffer, vortexed for 10 seconds, centrifuged at 13,000 g for 5 minutes and discarded the supernatant. Then, the bacterial pellet was suspended in 60 µl of 0.25X TE buffer and heated at 95°C for 10 minutes in a Thermal mixer (Eppendorf, Germany). Then, samples were vortexed for 20 seconds and cooled to room temperature, centrifuged at 13,000 g for 5 minutes and the supernatant which contained the crude DNA was transferred into a sterile 1.5 ml microcentrifuge tube and used as template in the PCR.

### Identification of DEC and DEC pathotypes

DEC pathotypes can be molecularly identified and/or classified based on the virulence genes specific to each pathotype 11. To identify the pathotypes, we used previously published PCR primers 14 targeting eleven virulence genes specific to six pathotypes i.e. ETEC – heat stable toxins (ST1 & ST2) and heat labile toxin (LT); EHEC – verotoxins (VT1, 2 & 2e); EIEC – Einv; EAEC - Eagg; EPEC – attaching and effacing antigen (eaeA); CDEC (Cell-detaching *E. coli*) – cytotoxic necrotising factors (CNF 1 & 2). Primer sequences used are described in Table S1 (Additional file 1) while the identification of DEC and DEC pathotypes is depicted in Figure S1 (Additional file 2).

The PCRs were prepared by following the Taq 2X Taq Master Mix protocol (New England BioLabs, Inc.). Briefly, a 25 µL PCR assay was prepared by mixing 12.5 µL of Taq 2X master mix, 1 µL each of the forward & reverse primers, 8 µL of nuclease free water, and 2.5 µL of crude DNA extract. Amplification was achieved in a thermocycler by using a programme reported by Todd et al. 15 with minor modifications i.e. Initial denaturation at 94°C for 5 minutes followed by 30 cycles of denaturation at 94°C for 60 seconds, annealing at 55°C for 90 seconds, extension at 72°C for 90 seconds, and a final extension step at 72°C for 10 minutes. Approx. 5 µL of the PCR product was analysed by agarose gel electrophoresis (2% w/v agarose) in Tris-acetate EDTA (TAE) buffer stained with Ethidium Bromide (5 mg/ml) (Sigma-Aldrich, USA). Gels were run at 120V for 1 hour and visualized with UVP Gel documentation system (Benchtop Trans-illuminator System BioDoc-it, UK). PCRs included negative controls (*E. coli* ATCC25922 & sterile nuclease free water) and positive controls (PDAS 101, ATCC 35401, 29930, 933W, 35150 & E 2348/69 from the Kenya Medical Research Institute).

Statistical analysis was performed with SPSS v23 and comparisons performed with the Chi square test to assess associations between variables, for which p-values less than 0.05 were considered statistically significant. Outcome variables were presence or absence of DEC and DEC pathotypes; predictor variables were age, sex, prior use of antibiotics and HAART (highly active antiretroviral therapy), temperature and HIV status.

## Results

### Demographics

Table 1 summarizes the participants' demographics. Of the 267 children with acute diarrhoea, 68 (25.5%) were HIV-positive while 199 (74.5%) were HIV-negative. The participants' age ranged from 6 to 59 months with a mean age of 29.622 ± 0.946 months and median age of 28 months; 154 (57.7%) children were male while 113 (42.3%) were female. Of the 68 HIV-positive children, 29 (42.6%) were male while 39 (57.4%) were female; 64 (94.1%) of the HIV-positive children were on HAART while 4 (5.9%) were not because they were newly diagnosed. Of the 199 HIV-negative children, 125 (62.8%) were male while 74 (37.2 %) were female. The modal age group were 13–24 months for HIV negative and 25–36 months for HIV-positive children.

**Table 1:** Demographics of the children with acute diarrhoea in Southern Uganda (n=267)

Age (months)	Sex		HIV status		On HAART	
	Male (%)	Female (%)	Positive (%)	Negative (%)	Yes (%, n=68)	No (%, n=68)
6–12	20	24	2	42	1	0
13–24	35	37	13	59	11	0
25–36	38	20	20	38	18	1
37–48	32	19	16	35	14	2
49–59	29	13	17	25	20	1
**Total**	**154** **(57.7)**	**113 (42.3)**	**68 (25.5)**	**199 (74.5)**	**64** **(94.1)**	**4 (5.9)**

Furthermore, 78 samples were screened by PCR for presence of HIV (for children who were less than 18 months), six of whom were positive (4 male, 2 female), Table 2. Most (66.7%, 4/6) of these children belonged to the age group 13–18 months.

**Table 2:** PCR screening of dried blood spots (DBS) for detection of HIV-1

Age (months)	Sex				Total		
	Male		Female				
	HIV+	HIV-	HIV+	HIV-	HIV+	HIV-	Overall
6–12	01	19	01	23	02	42	44
13–18	03	16	01	14	04	30	34
**Total**	**04**	**35**	**02**	**37**	**06**	**72**	**78**

Most children (61.4%, 164/267) had used antibiotics prior to visiting the hospital especially children in the 13 to 24-month age group. Relatedly, this age group had the highest number of children with a body temperature of above 37.5°C, Table 3. However, there was no association between the use of antibiotics and elevated body temperature (X2=0.030, df=1, p=0.862, p≥0.05).

**Table 3:** Previous use of antibiotics, body temperature and age category

Age group (months)	Prior use of antibiotics		Body temperature	
	Yes (%)	No (%)	36–37.5°C (%)	Above 37.5°C (%)
6–12	32	12	28	16
13–24	47	25	42	30
25–36	31	27	37	21
37–48	30	21	29	22
49–59	24	18	28	14
**Total**	**164 (61.4)**	**103 (38.6)**	**164 (61.4)**	**103 (38.6)**

### Prevalence of DEC and DEC pathotypes

Of the 267 children with acute diarrhoea, 38.2% (102/267) had DEC. The prevalence of DEC in HIV-positive children and HIV-negative children was 45.6% (31/68) and 35.7% (71/199), respectively, and the difference in prevalence was not statistically significant regarding HIV status (p=0.2662), Table 4. A total of six DEC pathotypes were detected; EAEC was the most frequent (14.2%) while CDEC was the least frequent (0.75%). Individually, ETEC was associated with HIV-negative status while EAEC and CDEC were associated with HIV-positive status, Table 4. Generally, the virulence genes were more frequent in DEC pathotypes from HIV-negative children compared to pathotypes from HIV-positive children but the difference was not significant (p=0.0662), Table 5. However, the CDEC CNF1 gene entirely occurred in HIV-positive children (p=0.015), Table 5. Besides the detection of DEC, other enteric bacteria as well as parasites were also detected, Table 6.

**Table 4:** Frequency of DEC pathotypes among children and association with HIV status

Pathotype	Prevalence among HIV+ (N=68), n (%)	Prevalence among HIV- (N=199), n (%)	Overall prevalence (N=267), n (%)	P-value
EAEC	15 (22.1)	23 (11.6)	38 (14.2)	**0.0324**
EPEC	8 (11.8)	10 (0.5)	18 (6.7)	0.0557
ETEC	0 (0)	16 (8)	16 (6)	**0.0159**
EIEC	6 (8.8)	14 (7)	20 (7.5)	0.6286
EHEC	0 (0)	8 (4)	8 (3)	0.0932
CDEC	2 (2.9)	0 (0)	2 (0.75)	**0.0152**
**Total**	**31 (45.6)**	**71 (35.7)**	**102 (38.2)**	0.2662

**Table 5:** Frequencies of virulence genes among DEC pathotypes and their relationship with HIV status

Pathotype	Gene	Frequency		X^2^ P- value

		HIV-positive (N=68), n (%)	HIV-negative (N=267), n (%)	
ETEC	*LT1*	0	9	0.744
	*ST1*	0	4	0.293
	*ST2*	0	3	0.309
EHEC	*VT1*	0	4	0.744
	*VT2*	0	6	0.148
	*VT2e*	0	0	-
CDEC	*CNF1*	2	0	**0.015**
	*CNF2*	0	0	-
**Total**		**2 (3)**	**26 (10)**	0.0662

**Table 6:** Other enterobacteria and parasites detected

Age (months)	DEC		Other pathogens detected			

	HIV+ (%, n=68)	HIV- (%, n=199)	Salmonella spp. (%)	*Shigella* spp. (%)	Hook worms (%)	*Trichomonas hominis* (%)
6–12	1	8	0	0	0	1
13–24	4	17	0	0	0	0
25–36	4	13	0	0	0	4
37–48	9	19	2	1	0	5
49–59	13	14	2	0	2	9
**Total**	**31 (45.6)**	**71** **(35.7)**	**4 (1.5)**	**1 (0.4)**	**2 (0.7)**	**19 (7.1)**

## Discussion

In this study, the overall prevalence of DEC was found to be high in Ugandan children with acute diarrhoea. As *E. coli* is a frequent cause of childhood diarrhoea in Uganda 12, DEC could be a significant problem that deserves more attention. Our prevalence for DEC (38.2%) was higher than the reported rate from Tanzania (22.9%)^16^ but lower than rates from Nigeria (73.8%) 6 and India (52%)^11^. However, regarding HIV status our findings are comparable to similar studies in Africa and other continents. For example, the exclusive detection of a DEC pathotype (i.e., CDEC) in HIV-positive cases has been reported before^17^. Similar to our findings, studies on DEC in East Africa show that EAEC is the most frequent pathotype among children with diarrhoea^18^–^23^; in Western Kenya, ETEC was also associated with acute diarrhoea while EAEC was linked to persistent diarrhoea in HIV-positive children^20^. Overall, studies elsewhere have shown variations in prevalence of DEC pathotypes in children^24^.

The distribution of virulence genes in DEC pathotypes in relation to the type of diarrhoea has been evaluated before. In Kenya, acute diarrhoea among HIV-positive cases was found to be associated with EAEC's aatA and EPEC's bfp, while EIEC's ipaH, EHEC's stx1/stx2 and ETEC's elt/est were associated with HIV-negative cases 20. In our study, CDEC's CNF1 was only detected in HIV-positive children but generally there was no significant association between detection of the genes and HIV status. Some of the differences observed between our findings and other works could be due to environmental factors, study design effects, differences in levels of hygiene, sanitation/sewage management, host susceptibility or unknown underlying health conditions but further studies are required to clearly elucidate the source of these variations.

Furthermore, Okeke et al 25 noted that CNF1 and CNF2 are frequently found in invasive *E. coli* isolates. Overall, the dual occurrence of multiple virulence genes as opposed to a single gene raises a question as to whether these genes act in synergy to produce acute disease^26^. Several studies indicate that *E. coli* pathotypes require multiple genes to be highly virulent 27. For instance, ETEC with heat-labile toxin elt, heat-stable toxin est and colonization factor antigens (CFAs); EPEC with bfp and eae genes and Shiga-toxin-producing *E. coli* (STEC or EHEC) with shiga-like toxin stx and eaeA, are considered the most virulent strains 28. Although EAEC strains are genetically heterogeneous and contain various virulence genes including aggR 29, the presence of multiple genes has not been associated with EAEC pathogenesis. This may explain why EAEC is predominantly associated with persistent diarrhoea while strains with multiple genes (i.e. EPEC, ETEC and EHEC) are associated with acute diarrhoea 30. In our study we detected multiple genes in ETEC (LT1, ST1, ST2) and EHEC (VT1, VT2) but we did not evaluate their association with acute diarrhoea. Lastly, in our study the 13 to 24-month age group had the highest number of children with a body temperature of ≥37.5°C however, we were not able to establish the cause of high body temperature though some children (38.6%, n=103) had a positive blood smear for malaria parasites. Relatedly, stool analysis showed coinfection with multiple pathogens in 10% (26/267) of the children, which complicates the aetiology of acute diarrhoea in these children. However, the coinfection rate (10%) in this study is lower than the 22.1% rate reported in India^11^.

## Conclusions

DEC is prevalent in Ugandan children with acute diarrhoea hence, DEC should be ruled-out by diagnostics targeting the aetiology of acute diarrhoea. The overall prevalence of DEC pathotypes in HIV-positive children vs. HIV-negative children was not statistically significant however, ETEC was associated with HIV-negative status while EAEC and CDEC were associated with HIV-positive status.

## List of abbreviations

**DEC:** Diarrhoeagenic *Escherichia coli*; EAEC: Enteroaggregative Escherichia coli; EPEC: Enteropathogenic Escherichia coli; ETEC: Enterotoxigenic *Escherichia coli*; EIEC Enteroinvasive *Escherichia coli*; EHEC: Enterohemorrhagic *Escherichia coli*; CDEC: Cell-detaching Escherichia coli

## Declarations

### Availability of data and materials

All data generated or analysed during this study are included in this published article and its supplementary information files.
